# Sociodemographic Factors and Prejudice toward HIV and Hepatitis B/C Status in a Working-Age Population: Results from a National, Cross-Sectional Study in Japan

**DOI:** 10.1371/journal.pone.0096645

**Published:** 2014-05-02

**Authors:** Hisashi Eguchi, Koji Wada, Derek R. Smith

**Affiliations:** 1 Department of Public Health, Kitasato University School of Medicine, Sagamihara, Kanagawa, Japan; 2 National Center for Global Health and Medicine, Tokyo, Japan; 3 School of Health Sciences, Faculty of Health, University of Newcastle, Ourimbah, Australia; Saint Louis University, United States of America

## Abstract

**Background:**

In many countries, HIV, hepatitis B virus (HBV), and hepatitis C virus (HCV) infected individuals may face discrimination and mistreatment from coworkers. Effective interventions to reduce workplace discrimination are therefore needed to protect these vulnerable populations. The current study investigated potential associations between sociodemographic factors and prejudice toward HIV and HBV/HCV infected colleagues within a Japanese working population.

**Methods:**

An online anonymous, nationwide internet survey was administered to a cross-section of approximately 3,000 individuals in Japan. The survey comprised 14 questions focusing on demographics (five items), basic HIV or HBV/HCV knowledge (eight items), and potential prejudice toward HIV or HBV/HCV infected colleagues (one item). The sociodemographic characteristics evaluated were sex, age, educational level, employment status, and individual income; with multiple logistic regression used for the analysis.

**Results:**

In total, 3,055 individuals were recruited for the HIV related survey and 3,129 for the HBV/HCV related survey. Older age was significantly and positively associated with prejudice toward HIV infected colleagues (p<0.01) and negatively associated with prejudice toward HBV/HCV infected colleagues (p<0.01). Statistically significant associations were not observed between other sociodemographic characteristics and potential prejudice toward HIV and HBV/HCV infected coworkers.

**Conclusion:**

Overall, this study suggests that age may be associated with prejudice toward HIV and HBV/HCV infected colleagues among the working age population of Japan. As such, policy makers should consider the age of participants when formulating efforts to reduce prejudice toward HIV and HBV/HCV infected workers.

## Introduction

Research suggests that individuals diagnosed with HIV or HBV/HCV may face abuse, discrimination, and unfair treatment from coworkers [1] [2]. Furthermore, employers are often reluctant to hire people diagnosed with HIV, and may even dismiss a newly-diagnosed employee [3]. As a result, seropositive workers often avoid disclosing their infection status to employers in order to avoid prejudice and disadvantage [4] [5].

HBV/HCV infection initially entered the general Japanese population through blood transfusions, acupuncture, tattooing, and skin injury [6]. The spread of HCV infection was caused primarily by viral-contaminated blood products; and approximately 10,000 cases of infection occurred in individuals who received these products up to 1988 [7]. In recent years, the horizontal incidence of HBV/HCV infection in Japan has decreased steadily from 1.43% to 0.10% in men, and from 0.95% to 0.03% in women; although HBV carriers are most numerous in the 50–59 year age group, and the estimated number of HCV carriers is highest in individuals older than 50 years [8].

Intervention programs intended to improve the general population's knowledge of HIV and HBV/HCV and reduce stigma have often been inconsistent [9]. Older age independently predicted a stronger discriminatory attitude in a study from Hong Kong [10] for example, while it decreased the likelihood of receiving HIV screening in a previous study from the United States (US) [11]. Cultural values and social-cognitive factors are also known to affect HBV screening intention [12].

To the authors' knowledge, no studies have yet explored the association between sociodemographic factors and prejudice toward HIV and HBV/HCV infected colleagues in Japanese working populations. As such, the current study was undertaken to examine associations between sociodemographic factors and prejudice toward HIV and HBV/HCV infected colleagues in this large demographic. We hypothesized that sociodemographic factors may effect prejudice toward HIV and HBV/HCV infected colleagues.

## Methods

### Participants and conduct of the survey

This study comprised a HIV related survey and a HBV/HCV related survey. Around 3,000 individuals of working age (between 20 and 69 years of age), without sex or age bias, were randomly extracted from the registry of an Internet research company (among a randomly selected group of 7,937 persons from a total of 1.60 million registrants), and a survey of this population was then carried out via the Internet in September 2011. A random number generator was used to select participants, with the study population itself comprising of individuals who were interested in participating a survey with financial incentives for responding. The web survey company contacted selected registrants to respond to the survey and ceased recruitment when the total number of participants reached the target number. Sample size calculation was based on an expected percentage of having prejudiced toward HIV and HBV/HCV infected colleagues of 40% with an expected confidence interval precision of ± 5% [13]. The minimum sample size required was 248 participants. Participants were recruited in clusters, targeting 300 individuals for each sex in each age group. The survey consisted of 14 questions on each participant's sociodemographic characteristics (five items), basic knowledge of factors affecting HIV or HBV/HCV (eight items), and potential prejudice toward HIV or HBV/HCV infected colleagues. The sociodemographic characteristics investigated were sex, age, educational level, employment status, and individual income.

### Participant characteristics

Age was stratified into five groups: 20–29, 30–39, 40–49, 50–59, and 60–69 years. Educational level was divided into four categories as follows: lower than or equal to high school graduation; technical college or junior college; higher than university; and others. Employment status was classified into five groups: manager, regular employee, non-regular employee, others, and student. The other employment status included: unemployment, agriculture, fishery, forestry, and self-employed business owners. Annual individual income was classified in three equal groups as follows: low, <1 million yen (<12,500 US$); middle, 1–3 million yen (12,500–24,999 US$); and high, >3 million yen (≥25,000 US$) (with an exchange rate of 1 US$ equaling around 80 Japanese yen at the time).

### HIV or HBV/HCV knowledge and prejudice toward infected colleagues

Eight questions were developed based on previous studies, with responses measured on a two-point scale (0 =  No, I did not know, or 1 =  Yes, I knew). Cronbach's alpha was 0.82 for the HIV related survey and 0.92 for the HBV/HCV related survey. The sum of each item was calculated, with the higher score suggesting greater knowledge on HIV and HBV/HCV. Prejudice toward HIV and HBV/HCV infected individuals was determined based on the response to the following question: “If I found that people with whom I work were infected with HIV or HBV/HCV, I think I would look at him/her to be a homosexual, someone who engaged in sexual relationships with an unspecified number of people, or a drug addict.”

Participant responses toward HIV infected individuals were initially measured on a five-point scale (1 = I think so; 2 = I think so, to a degree; 3 = I do not really think so; 4 = I do not think so at all; and 5 =  not sure), and then further dichotomized into a two-point scale (1 = I think so and I think so to a degree; and 0 = I do not really think so and I do not think so at all). The “Not sure” (5) response on the initial five-point scale (n = 230) was omitted during analysis to allow for a direct comparison between results from the HIV and the HBV/HCV related surveys. Responses to the HBV/HCV related survey were initially measured on a four-point scale (1 = I think so; 2 = I think so to a degree; 3 = I do not really think so; and 4 = I do not think so at all), and then further dichotomized into a two-point scale (1 = I think so and I think so to a degree; and 0 = I do not really think so and I do not think so at all).

### Statistical analysis

Multiple logistic regression analyses were used to investigate the associations between each sociodemographic factors and prejudice toward HIV and HBV/HCV infected colleagues. Given that the outcome of interest was not rare, we subsequently applied Zhang's formula of adjustment when calculating the results [14]. Statistical analysis was performed using SPSS version 17.0 (IBM, Chicago, IL, USA). A two-tailed p value of 0.05 was considered significant, unless otherwise indicated.

### Ethics

The study aims and protocol were approved by the institutional ethics committee of Kitasato University School of Medicine in 2011. Participants were informed in advance that their participation was strictly voluntary and that all information provided would remain confidential. Individuals who consented to participate in the survey were able to access a designated website upon verification of their personal information, after which time they could then complete the survey online. Participants had the option to not respond to any part of the questionnaire at any time, and also the option to discontinue the survey at any point. Consent was implied based on voluntary participation according to the ethics code for public health research in Japan [15].

## Results

The survey was sent to 7,937 individuals and closed after each stratum of gender and age group reached its target sample size. Recruitment was terminated when the number of participants reached 3,055 in the HIV related survey and 3,129 in the HBV/HCV related survey. Each of the five age groups (20–29, 30–39, 40–49, 50–59, and 60–69 years old) contained approximately 20% of the total participants. Participant characteristics are summarized in Table 1. Educational level distribution was significantly different between participants of the HIV and HBV/HCV related surveys (p<0.01). Mean HIV knowledge score was higher than the mean HBV/HCV knowledge score (p<0.01). Participants answering, “strongly agree” and “agree” about prejudice toward infected colleagues constituted 39.8% and 23.7% of total participants in the HIV and HBV/HCV related surveys, respectively.

**Table 1:** Participant characteristics.

	HIV		HBV/HCV		p
	n = 3,055	(%)	n = 3,129	(%)	
Sex					
Male	1,532	(50.1)	1,549	(49.5)	0.61
Female	1,523	(49.9)	1,580	(50.5)	
Age (yr)					
20–29	607	(19.9)	618	(19.8)	1.00
30–39	611	(20.0)	628	(20.1)	
40–49	612	(20.0)	627	(20.0)	
50–59	616	(20.2)	632	(20.2)	
60–69	609	(19.9)	624	(19.9)	
Educational level					
Junior high school or high school	878	(28.7)	693	(22.1)	<0.01
Technical college or junior college	767	(25.1)	572	(18.3)	
University and graduate school	1,368	(44.8)	1,084	(34.6)	
Others	42	(1.4)	780	(24.9)	
Employment status					
Manager	244	(8.0)	231	(7.4)	0.78
Regular employee	852	(27.9)	845	(27.0)	
Non-regular employee	522	(17.1)	540	(17.3)	
Undergraduate student	201	(6.6)	211	(6.7)	
Others	1236	(40.5)	1302	(41.6)	
Individual income					
Low (<1 million yen/year)	1,210	(39.6)	1,236	(39.5)	0.07
Middle (1–3 million yen/year)	1,052	(34.4)	1,149	(36.7)	
High (>3 million yen/year)	793	(26.0)	744	(23.8)	
Having prejudiced opinions about infected colleagues					
Strongly agree and agree	1,215	(39.8)	742	(23.7)	-
Disagree and strongly disagree	1,610	(52.7)	2,387	(76.3)	
Not sure	230	(7.5)	-	-	
	Mean	SD	Mean	SD	
Knowledge score (0–8)	6.64	1.91	5.33	2.94	<0.01

Table 2 summarizes the associations between sociodemographic factors and prejudice toward infected colleagues. The age and educational level distributions were significantly different in the HIV related survey (p<0.01, 0.03 respectively). The HIV knowledge score of “strongly agree” and “agree” respondents was also lower than that observed among the “disagree and strongly disagree” respondents (p<0.01). Sex, age, employment status, and individual income distributions were significantly different in the HBV/HCV related survey (p<0.01, 0.01, 0.01, and 0.04, respectively). The HBV/HCV knowledge score of “strongly agree” and “agree” respondents was lower than observed in the “disagree” and “strongly disagree” respondents (p<0.01).

**Table 2:** Statistical associations between socio-demographic factors and prejudiced attitudes towards infected colleagues.

	Prejudiced toward infected HIV colleagues (n = 3,055)							Prejudice toward infected HBV/HCV colleagues (n = 3,129)				
	Strongly agree and agree	(%)	Disagree and strongly disagree	(%)	Not sure	(%)	p	Strongly agree and agree	(%)	Disagree and strongly disagree	(%)	p
Sex												
Male	629	(41.1)	790	(51.6)	113	(7.4)	0.35	409	(26.4)	1140	(73.6)	<0.01
Female	586	(38.5)	820	(53.8)	117	(7.7)		333	(21.1)	1247	(78.9)	
Age												
20–29	209	(34.4)	343	(56.5)	55	(9.1)	<0.01	174	(28.2)	444	(71.8)	0.01
30–39	226	(37.0)	342	(56.0)	43	(7.0)		160	(25.5)	468	(74.5)	
40–49	238	(38.9)	323	(52.8)	51	(8.3)		142	(22.6)	485	(77.4)	
50–59	258	(41.9)	320	(51.9)	38	(6.2)		125	(19.8)	507	(80.2)	
60–69	284	(46.6)	282	(46.3)	43	(7.1)		141	(22.6)	483	(77.4)	
Educational level												
Junior high school or high school	347	(39.5)	451	(51.4)	80	(9.1)	0.03	150	(21.6)	543	(78.4)	0.52
Technical college or junior college	304	(39.6)	410	(53.5)	53	(6.9)		139	(24.3)	433	(75.7)	
University and graduate school	551	(40.3)	728	(53.2)	89	(6.5)		267	(24.6)	817	(75.4)	
Others	13	(31.0)	21	(50.0)	8	(19.0)		186	(23.8)	594	(76.2)	
Employment status												
Manager	101	(41.4)	130	(53.3)	13	(5.3)	0.06	59	(25.5)	172	(74.5)	<0.01
Regular employee	344	(40.4)	445	(52.2)	63	(7.4)		240	(28.4)	605	(71.6)	
Non-regular employee	195	(37.4)	287	(55.0)	40	(7.7)		107	(19.8)	433	(80.2)	
Others	516	(41.7)	626	(50.6)	94	(7.6)		283	(25.1)	1019	(74.9)	
Student	59	(29.4)	122	(60.7)	20	(10.0)		53	(21.7)	158	(78.3)	
Individual income (JPY)												
Low (<1 million yen/year)	465	(38.4)	640	(52.9)	105	(8.7)	0.20	264	(21.4)	972	(78.6)	0.04
Middle (1–3 million yen/year)	415	(39.4)	565	(53.7)	72	(6.8)		285	(24.8)	864	(75.2)	
High (>3 million yen/year)	335	(42.2)	405	(51.1)	53	(6.7)		193	(25.9)	551	(74.1)	
	Mean	SD	Mean	SD	Mean	SD	p	Mean	SD	Mean	SD	p
Knowledge score (0–8)	6.46	(1.85)	6.92	(1.71)	5.66	(2.89)	<0.01	4.76	(3.09)	5.51	(2.87)	<0.01

Logistic regression analyses revealed that older age was significantly associated with increased prejudice toward HIV infected colleagues (p<0.01) and decreased prejudice toward HBV/HCV infected colleagues (p<0.01), after adjusting for other sociodemographic factors and basic knowledge (Table 3). Sex, educational level, employment status, and individual income were not significantly associated with prejudice toward HIV or HBV/HCV infected colleagues.

**Table 3:** Univariable and multivariable analyses of association between sociao-demographic factors and the prejudiced attitudes toward HBV/HCV or HIV infected colleagues.

	Prejudiced toward infected HIV colleagues (n = 2,825)				Prejudice toward infected HBV/HCV colleagues (n = 3,129)			
	Univariable model^a^		Multivarible model^b^		Univariable model^a^		Multivarible model^b^	
	OR (95% CI)	p	OR (95% CI)	p	OR (95% CI)	p	OR (95% CI)	p
Sex								
Male	ref		ref		ref		ref	
Female	0.94 (0.85–1.02)	0.16	0.93 (0.83–1.05)	0.25	0.79 (0.68–0.90)	<0.01	0.87 (0.73–1.03)	0.14
Age								
20–29	ref		ref		ref		ref	
30–39	1.05 (0.91–1.20)	0.51	0.99 (0.83–1.15)	0.85	0.90 (0.74–1.09)	0.29	0.80 (0.63–1.00)	0.06
40–49	1.11 (0.97–1.25)	0.12	1.04 (0.88–1.20)	0.65	0.79 (0.64–0.97)	0.03	0.70 (0.54–0.90)	0.01
50–59	1.16 (1.02–1.29)	0.02	1.08 (0.93–1.24)	0.31	0.68 (0.54–0.85)	0.00	0.61 (0.46–0.81)	0.00
60–69	1.24 (1.13–1.35)	<0.01	1.16 (1.01–1.30)	0.03	0.79 (0.64–0.97)	0.03	0.70 (0.52–0.92)	0.02
Test for linear trend^d^	p<0.01		p<0.01		p<0.01		p<0.01	
Educational level								
Junior high school or high school	ref		ref		ref		ref	
Technical college or junior college	0.98 (0.86–1.10)	0.72	1.02 (0.90–1.14)	0.34	1.12 (0.92–1.34)	0.26	1.16 (0.95–1.40)	0.13
University and graduate school	0.99 (0.89–1.09)	0.86	1.05 (0.94–1.17)	0.64	1.13 (0.96–1.33)	0.15	1.17 (0.98–1.37)	0.08
Others	0.87 (0.52–1.31)	0.55	0.90 (0.53–1.36)	0.85	1.10 (0.91–1.31)	0.32	1.12 (0.93–1.34)	0.24
Test for linear trend^d^	p = 0.79		p = 0.60		p = 0.31		p = 0.21	
Employment status								
Manager	ref		ref		ref		ref	
Regular employee	1.00 (0.83–1.17)	0.97	1.07 (0.90–1.25)	0.44	1.11 (0.87–1.37)	0.39	1.04 (0.80–1.33)	0.74
Non-regular employee	0.92 (0.75–1.11)	0.41	1.01 (0.80–1.24)	0.94	0.76 (0.56–1.03)	0.08	0.78 (0.53–1.10)	0.15
Others	0.71 (0.51–0.95)	0.02	0.79 (0.53–1.11)	0.18	0.98 (0.70–1.33)	0.92	0.88 (0.56–1.32)	0.57
Student	1.03 (0.88–1.19)	0.68	1.07 (0.88–1.26)	0.51	0.84 (0.64–1.09)	0.20	0.88 (0.63–1.20)	0.46
Individual income (JPY)								
Low (<1 million yen/year)	ref		ref		ref		ref	
Middle (1–3 million yen/year)	1.01 (0.90–1.11)	0.90	0.93 (0.81–1.06)	0.31	1.15 (1.00–1.32)	0.05	1.11 (0.92–1.32)	0.24
High (>3 million yen/year)	1.07 (0.97–1.17)	0.18	0.98 (0.82–1.14)	0.77	1.20 (1.03–1.38)	0.02	1.04 (0.81–1.31)	0.65
Test for linear trend^d^	p = 0.20		p = 0.79		p = 0.01		p = 0.31	
Knowledge score	0.87 (0.83–0.90)	<0.01	0.87 (0.84–0.91)	<0.01	0.92 (0.90–0.95)	<0.01	0.93 (0.90–0.96)	<0.01

a Each factor was entered into the univariable model separately.

b All factors were entered into the multivariable model simultaneously.

c ref: Reference category OR: odds ratio.

d Test for linear trends were performed by modelling the group scores of age, educational level and individual income as one variable.

## Discussion

In the current study we investigated associations between sociodemographic factors and prejudice toward HIV and HBV/HCV infected colleagues among the Japanese working age demographic for what appears to be the first time. Our findings suggest that older age was independently associated with increased prejudice toward HIV infected colleagues and decreased prejudice toward HBV/HCV infected colleagues, after controlling for other sociodemographic factors and knowledge level. No other associations between other sociodemographic factors and these attitudes were elucidated in the current study. These results suggest that health promotion authorities should carefully consider the target population age when implementing interventions to reduce prejudice toward HIV and HBV/HCV infected colleagues in the workplace in Japan, as elsewhere.

It is reasonable to hypothesize that age reflects an individual's historical background, and therefore, may play an important contextual role in their reaction toward HIV. Our current findings are consistent with previous research which found that older age was independently associated with increased prejudice toward HIV infected colleagues [10] [16]. Individuals older than 40 years of age experienced the so-called ‘AIDS panic’ of January of 1987, which was caused by media reports of a Japanese woman in Kobe who had been diagnosed with AIDS, and died soon after the initial report. It has been suggested that this particular panic rose from a societal concern that the government was unable to prevent HIV from entering Japan [17]. From this the resulting panic sharply increased negative information about HIV in the Japanese mass media [18]. Once disseminated, this biased information led to discrimination against HIV infected people. As such, this unfortunate historical episode illustrates the need to consider the age of the target population to effectively reduce discrimination toward HIV infected colleagues in the workplace.

Older people in Japan may have different attitudes towards HBV/HCV infection, resulting from different initial exposure to HBV/HCV related information, when compared with younger people. The current study revealed that older age was independently associated with decreased prejudice toward HBV/HCV infected colleagues. It is worth noting that HCV hepatitis, when caused by HCV-contaminated fibrinogen concentrate, is termed Yakugai-hepatitis in Japan; where the characters Yakugai mean “health hazards due to pharmaceuticals” [7]. Until 1988, approximately 10,000 individuals who had received HCV-contaminated fibrinogen concentrate were diagnosed with HCV infection in Japan [7]. As a result, it is reasonable to assume that Yakugai-hepatitis positive individuals in Japan are more commonly perceived as victims, at least by the older generation. Since 1988, blood donated at Japanese blood centers has been screened for HBV, and HBV vaccinations have also been administered to infants born to HBV infected mothers. By contrast, most acute hepatitis B cases in contemporary Japanese society result from sexual transmission between young people [19] [20] [21]. Younger people in Japan may therefore perceive HBV infection as the responsibility of the individual and something that is acquired through immoral behavior. Thus, any intervention to decrease prejudice toward HBV/HCV infected colleagues will clearly need to consider the age of the target population in its design.

The mean HIV knowledge score in the current study was higher than that observed for HBV/HCV, which is consistent with previous research [22]. Since establishing government resources for medical and public health support of HIV infected people in Japan, the stigma surrounding HIV has diminished significantly over time [22]. Increased knowledge of HIV or HBV/HCV has previously been shown to reduce prejudice toward infected colleagues [23] [24]. Lessons may therefore, be learned from successful HIV public health interventions to improve the knowledge about HBV/HCV. Japanese public health centres/municipal health centres represent an existing on-the-ground resource for community health promotion [25] and may therefore be well-placed to serve an increasing workplace role in this regard.

Sex, educational level, employment status, and individual income were not independently associated with prejudice toward infected colleagues in the present study. This is somewhat different to other research from Western and African regions which demonstrated strong associations between sociodemographic factors, stigma, and discrimination against people living with HIV [26] [27] [28]. Cultural values and social-cognitive factors are related to health beliefs [12], and these are known to influence the perceptions of blood-borne diseases in other parts of Asia, such as China [29]. In a Japanese setting, at least in the current study, the effect of sociodemographic factors on prejudice toward HIV or HBV/HCV infected colleagues may be limited.

Although it appears to have been the first of its kind, there are several potential limitations to the current study. Firstly, 230 respondents who answered “not sure” on having prejudiced opinions about infected colleagues were omitted from the analysis, as previously described. The distribution of sex, age, employment status, individual income and having prejudiced opinions about infected colleagues among this omitted group was not significantly different from the remaining respondents. However, the proportion of “junior high school or high school” and “others” in the educational level category were found to be higher, while the proportion of “technical college or junior college” and “university and graduate school” were lower among the omitted group. Their knowledge score was also lower among the omitted group. As a result, the potential effect of excluding this particular group on the overall results may still be considered. Secondly, our study population presumably had internet access in order to complete the survey, and therefore may be more aware of HIV and HBV/HCV through access to online information [30]. Our results may therefore not be generalized to individuals without internet access, or in other countries and settings. Thirdly, our study population were higher educated and with lower income than the general Japanese working population; with only 25% of respondents having completed junior high or high school compared with 46% among the general Japanese working population [31] and 40% of respondents having an income of less than 1 million yen compared with 8.6% of that [32]. Fourthly, we could not be entirely certain about individuals who stated their educational category as ‘others,’ given that this could include college and/or high school drop-outs or simply participants who did not want to answer the question. Finally, although HBV and HCV are distinct diseases with different characteristics, different modes of transmission, and have different therapeutic options and goals; we did not measure prejudiced toward these diseases separately.

## Conclusion

Overall, this study suggests that age may be associated with prejudice toward HIV and HBV/HCV infected colleagues among the working age population of Japan. As such, policy makers should carefully consider the age of participants when formulating efforts to reduce prejudice toward HIV and HBV/HCV infected workers.
